# Possibilities of Interpreting the Night-to-Day Ratio Specified by 24-Hour Blood Pressure Monitoring

**DOI:** 10.1155/2023/6530295

**Published:** 2023-01-31

**Authors:** Alena Havelkova, Petr Dvorak, Jarmila Siegelova, Petr Dobsak, Petr Filipensky, Germaine Cornelissen

**Affiliations:** ^1^Department of Physiotherapy, Faculty of Medicine, Masaryk University, Brno, Czech Republic; ^2^St. Anne's Teaching Hospital, Brno, Czech Republic; ^3^Department of Biology, Faculty of Veterinary Hygiene and Ecology, University of Veterinary Sciences, Brno, Czech Republic; ^4^Halberg Chronobiology Center, University of Minnesota, Minneapolis, MN, USA

## Abstract

**Aim:**

Specify the risk rate of incorrect patient classification based on the night-to-day ratio specification from singular 24-h ABPM in comparison to the results of 7-day ABPM monitoring

**Materials and Methods:**

1197 24 h cycles were enrolled in 171 subjects in the study and divided into 4 groups: group 1 (40 healthy men and women without exercise), group 2 (40 healthy exercise-training men and women), group 3 (40 patients with ischemic coronary artery disease without exercise), and group 4 (51 patients with ischemic coronary artery disease following cardiovascular rehabilitation). The subject of the evaluation was the percentage rate of incorrect subject classification (dipper, nondipper, extreme dipper, and riser) based on the mean blood pressure values for 7 days and from seven independent 24-hour cycles (the mean value mode).

**Results:**

In the case of the individuals included in the monitored groups, the mean night-to-day ratio-based (mode for the 7 days versus the individual days of 24-hour monitoring) classification accordance ranged between 59% and 62%. Only in singular cases did the accordance reach 0% or 100%. The accordance size was not dependent on the health or cardiovascular disease (*p* < 0.594; 56% vs. 54%) or physical activity (*p* < 0.833; 55% vs. 54%) of the monitored individuals.

**Conclusion:**

The specification of the night-to-day ratio of each individual for each day of the 7-day ABPM monitoring would be the most convenient option. In many patients, diagnosing could thus be based on the most frequently occurring values (mode specification).

## 1. Introduction

Although blood pressure measurement in outpatient care is standardised and governed by accurately defined principles, there is a statistically significant probability of erroneous hypertension diagnosis [1, 2]. Other methods, including 24-h ambulatory blood pressure monitoring (ABPM), are not yet globally used in hypertension diagnosis and treatment [3–5]. Physiological blood pressure decrease during sleep in comparison to the awake state should be between 10 and 20% in healthy individuals-dippers (D) [6]. Unusual conditions include sleep blood pressure decrease by more than 20%. Individuals with this condition are called extreme dippers (ED) and are threatened by the risk of insufficient perfusion of the vital organs. Repeated identification of the higher incidence of silent cerebral infarct and negative prediction of stroke are connected with patients classified as extreme dippers [7]. On the other hand, individuals not reaching even a 10% blood pressure drop during sleep are called nondippers (ND). This abnormal condition occurs in 25% of essential hypertonic patients [8]. The connection between the nondipper profile and hypertension may have fatal consequences for the whole cardiovascular system. Characteristics include a higher incidence of organ failures, retinopathy, left ventricle hypertrophy, or the risk of the cognitive deficit as a consequence of a past silent cerebrovascular incident. The nondipper condition is causally related to sympathetic nervous system pathology [8]. The occurrence of the nondipper profile multiplies with increasing age [9]. Numerous studies confirm high percentages of nondippers among patients with sleep disorders, especially in the case of narcoleptics with cataplexy in combination with rapid eye movement sleep stage deregulation [10]. The last group is represented by reverse dippers (called risers), with an opposite situation, i.e., mean blood pressure values are lower in daytime than at night. The “risers” condition appears in individuals working night shifts. From the predictive point of view, individuals with the reverse profile are most prone to cardiovascular incidents [11, 12]. For example, nocturnal reverse blood pressure dipping is a typical feature of cardiovascular dysautonomia accompanying Parkinson's disease [13].

Most authors base the previous classification according to the night-to-day ratio solely on 24-hour blood pressure monitoring. Night-to-day ratio may be specified by evaluating the mean blood pressure values from the seven monitored days as well as by evaluating the individual 24-hour cycles [14]. The purpose of this study was to specify the risk rate of incorrect patient classification based on night-to-day ratio specification from singular 24-h ABPM in comparison to the results of 7-day continuous ABPM, to compare healthy individuals with patients with an already diagnosed cardiovascular disease, and to prove the existence of a potential relationship (if any) to the gender of the monitored individuals or to the type of physical activity.

## 2. Methods

In total, 1197 24-h cycles were enrolled in 171 subjects in the study and divided into 4 groups. The first group included 40 subjects (20 men and 20 women) (mean age 26 ± 5.2 yrs; mean BMI 23.9 ± 3.61) without chronic medication. The second group of healthy exercise-training individuals included 20 men and 20 women (mean age 29 ± 7.8 yrs and mean BMI 22.3 ± 5.18) without chronic medication. The third group included 40 patients with chronic ischemic coronary artery disease (20 men, 20 women, mean age 67 ± 9.8 yrs, mean BMI 30.6 ± 7.22, and mean left ventricle ejection fraction 61 ± 8.2%). The fourth group included 51 patients with chronic ischemic coronary artery disease (39 men, 12 women, mean age 62 ± 8.9 yrs, mean BMI 32.7 ± 5.18, and mean left ventricle ejection fraction 59 ± 9.5%) following cardiovascular rehabilitation. The first part of the study involved evaluating the 40 healthy individuals of the first group without chronic medication and 40 patients (group 3) with chronic ischemic coronary artery disease without exercise. The second part of the study focused on assessing night-to-day ratio changes in the healthy individuals following controlled combined aerobic-resistance exercise (group 2) and in the patients with chronic ischemic coronary artery disease (group 4) following cardiovascular rehabilitation. All patients (third and fourth groups) were treated with beta-blockers, ACE inhibitors, and statins. They were symptomatically stable and their medication was not changed during rehabilitation or during ambulatory monitoring.

### 2.1. Measurement Methods

The ambulatory monitoring (7-day ABPM) was performed with ambulatory blood pressure monitoring devices TM–2430 (A&D; Tokyo, Japan) based on both the cuff-oscillometric and Korotkoff sound method. The device automatically recorded all the regular measurements for the period of continuous seven days, between 6:00 a.m. and 10:00 p.m. every 30 minutes and between 10:00 p.m. and 6:00 a.m. every 60 minutes. Such short intervals are necessary to obtain a sufficient number of representative measurements. The monitored individuals recorded their daily activities, their sleep times, their subjective problems, and their pharmaceutical intakes throughout the monitoring period. To make the blood pressure values objectively comparable for all tested individuals, the time interval in which the blood pressure values corresponded to the sleep/awake times needed to be defined. For that purpose, the mean nocturnal blood pressure value for individual days was calculated for the time interval between 1:00 and 6:00 a.m., and the daily blood pressure means for the time interval between 10:00 a.m. and 10:00 p.m. Blood pressure values for the periods between 7:00 and 9:00 a.m. and 10:00 and 12:00 p.m. were excluded from night-to-day ratio calculation for the purpose of data standardisation. This way, the time intervals for blood pressure recording corresponding to the night sleep and day awakening periods were unified for all individuals enrolled.

### 2.2. Intervention

Combined aerobic-resistance exercise for 40 healthy individuals (group 2) was scheduled twice weekly in the form of 60-minute workouts with a load intensity at the level of submaximum heart frequency (75–85% SF_max_). The minimum time span between the workouts was 24 hours. The workout consisted of a 10-minute warm-up, 25 minutes of aerobic exercise (spinning bicycle or bicycle ergometer), 15 minutes of resistance exercise on gym machines (bench press, leg extension and pull down) with 40–50% 1-RM (one repetition maximum) intensity (3 series of 10 exercises), and a 10-minute cool-down (stretching of the main muscle groups). The cardiovascular exercise (group 4) took the form of sixty-minute workouts scheduled two to three times weekly. The nature of the workouts was similar, combined aerobic-resistance exercise. The workout intensity was set at the level of the first ventilation threshold (VAT-1), 666 corresponding to the following workout parameters: load (W) and heart rate (HR) [15, 16]. The resistance exercise intensity was specified by the 1-RM (one repetition maximum) method. The maximum load lifted across the full range of motion for every exercise was used as the benchmark for the workload intensity specification, corresponding to 30–60% 1-RM.

### 2.3. Statistical Analysis

The arithmetic mean was calculated for every hour from all measured systolic blood pressure (SBP) and diastolic blood pressure (DBP) values obtained by the 7-day monitoring, plus weighed means from the individual 24-hour cycles and the whole 7-day interval. The night-to-day ratio was calculated using the following formula: 100 × (SBP_day_ − SBP_night_)/SBP_day_ [6]. Each study subject was categorised according to the above-defined classification criteria in correspondence to the 7-day blood pressure profile: dippers (D), nondippers (ND), extreme dippers (ED), and reverse dippers (risers-R). At the same time, the night-to-day ratio profile percentages in the individual 24-hour cycles were calculated. Night-to-day ratio was evaluated both on the basis of the mean blood pressure values for the 7 days and on the basis of the seven separate 24-hour cycles obtained in the course of the 7-day ABPM (mode was used as the median value, i.e., the most frequent occurrence category). After that, the percentage of correct classifications based on the 24-hour monitoring in comparison to the 7-day monitoring was specified (shown in the tables as the accordance percentage). To compare the mean accordance differences (%) between groups, the two-sample *t*-test assuming unequal variances was used. *P* value = 0.05 was considered as statistically significant. Differences between the percentage of accordance of the overall mean for 7 days and the percentage of accordance of the mode were tested by paired *t*-test.

## 3. Results

Table 1 shows the percentage quotient of the same night-to-day ratio for each day and the mean night-to-day ratio calculated from the mean blood pressure values from the 7-day blood pressure monitoring in the group of 40 healthy men and women (group 1). However, extensive night-to-day ratio variability is visible here, shown by the percentage quotients values for the individual 24-hour profiles corresponding to the individual days of the week. The accordance size (in %) in the individual healthy subjects (men and women) is also shown in Table 1. The mean accordance in group classification pursuant to the night-to-day ratio based on the 24-hour monitoring in comparison to the 7-day monitoring is 55%, with the minimum at 29% and the maximum at 86%. The evaluation of the 7-day monitoring in comparison to the mean results for the 7 days and mode specification (the most frequent result) shows individual day congruence in 12 men (60%) a u 13 women (65%), the difference in 6 men (30%) and in 5 women (25%), and the nonspecification of a clear mode in 2 men (10%) and in 2 women (10%). The mean accordance in group (Table 1) classification pursuant to night-to-day ratio based on the mode of the 7-day in comparison to the 7-day monitoring is 62%, with the minimum at 43% and the maximum at 86%. The mode provides a significantly higher percentage of accordance than the mean of 7 days (*p* < 0.0017, 62% vs. 55%).

Table 2 shows the night-to-day ratio in the group of healthy exercise-training subjects (group 2). The evaluation of the 7-day monitoring in comparison to the mean results for the 7 days and mode specification (the most frequent result) shows individual day congruence in 17 men (42.5%), the difference in 1 man (2.5%) and the nonspecification of a clear mode in 2 men (5%) The evaluation of the 7-day monitoring in comparison to the mean results for the 7 days and mode specification for the individual 24-hour cycles shows congruence in 13 healthy women (32.5%), the difference in 3 women (7.5%), and the nonspecification of a clear mode in 4 women (10%). The accordance size in the individual healthy men and women under combined aerobic-resistance exercise is also shown in Table 2. The mean accordance in group classification pursuant to night-to-day ratio based on the 24-hour monitoring in comparison to the 7-day monitoring is 56%, however, with the minimum 0% accordance and the maximum 100% accordance. This 0% accordance in the monitored healthy individual no. 24, showing the riser result in 4 days and the dipper profile in the remaining 3 days, points to the risk of completely erroneous classification on the basis of mere 24-hour monitoring. The evaluation of the 7-day monitoring in comparison to the mean results in the individual 24-hour cycles for the 7 days, and the mode specification for the individual days shows congruence in 30 individuals (75%), the difference in 4 individuals (10%), and the nonspecification of a clear mode in 6 individuals (15%). The mean accordance in group (Table 2) classification pursuant to night-to-day ratio based on the mode of the 7-day in comparison to the 7-day monitoring is 60%, with the minimum at 43% and the maximum at 100%. The difference between accordances (56% vs. 60%) is not significant (*p* < 0.062).

Table 3 shows the percentage quotient of the same night-to-day ratio in the group of 40 patients with chronic ischemic coronary artery disease without exercise. The mean accordance in group classification pursuant to night-to-day ratio based on the 24-hour monitoring in comparison to the 7-day monitoring is 53%, however, with a minimum of 29% accordance and a maximum of 86% in accordance. The evaluation of the 7-day monitoring in comparison to the mean results in the individual 24-hour cycles for the 7 days and the mode specification for the individual days shows congruence in 26 individuals (65%), a difference in 9 individuals (22.5%), and the nonspecification of a clear mode in 5 individuals (12.5%). The mean accordance in group (Table 3) classification pursuant to night-to-day ratio based on the mode of the 7-day in comparison to the 7-day monitoring is 59%, with the minimum at 43% and the maximum at 86%. The mode provides a significantly higher percentage of accordance than the mean of 7 days (*p* < 0.007, 59% vs. 53%).

Table 4 shows the percentage quotient of the same night-to-day ratio in the 51-subject cohort of patients with chronic ischemic coronary artery disease (group 4) under controlled combined exercise. The mean accordance in group classification pursuant to night-to-day ratio based on the 24-hour monitoring in comparison to the 7-day monitoring is 54%. The night-to-day ratio-based categorisation accordance variation span ranges from 14 to 100%. This cohort included 3 individuals (6%) for whom the 24-hour monitoring would be absolutely sufficient. This represents a difference in comparison to the monitored individuals from the previous groups-120 subjects in total (Tables 1–3), except for one case (Table 2). The evaluation of the 7-day monitoring of the 51 patients in comparison to the mean results for the 7 days and mode specification for the individual days (24-hour cycles) shows congruence in 32 patients (63%), difference in 8 patients (16%), and the nonspecification of a clear mode in 11 patients (21%). The mean accordance in group (Table 4) classification pursuant to night-to-day ratio based on the mode of the 7-day in comparison to the 7-day monitoring is 59%, with the minimum at 29% and the maximum at 100%. The difference between accordances (59% vs. 54%) is significant (*p* < 0.004).

The difference in the accordance size between the exercising subjects (healthy men and women and patients under cardiovascular rehabilitation) (Tables 2 and 4) and subjects without exercise (Tables 1 and 3) was not statistically significant (*p* < 0.833; 55% vs. 54%). The difference in the accordance size between young healthy individuals (mean age 27, 5 yrs; Tables 1 and 2) and patients with cardiovascular disease (mean age 64 yrs; Tables 3 and 4) was not statistically significant (*p* < 0.594; 56% vs. 54%). The difference in the accordance size between patients without exercise (Table 3) and patients under cardiovascular rehabilitation (Table 4) was not statistically significant (*p* < 0.783; 53% vs. 54%).

In addition, in the case of the comparison between the group of healthy individuals without physical activity (Table 1) and the group of healthy individuals under combined aerobic-resistance exercise (Table 2), no statistically significant difference was found (*p* < 0.818; 55% vs. 56%) in the night-to-day ratio-based categorisation accordance rate as a consequence of the performed physical activity.

## 4. Discussion

There are studies pointing to ethnic differences in blood pressure response to physical activity [17–19]. Likewise, the literature references contain proof of stronger blood pressure response in females [20, 21]. This is also confirmed by the present study finding higher nondipper (ND) and extreme dipper (ED) rates in healthy women in comparison to healthy men (Tables 1 and 2). However, the present study focused above all on the identification of the possible risk of incorrect subject classification as dipper, nondipper, extreme dipper, or reverse dipper based on singular 24-hour blood pressure monitoring. The first test was performed in two cohorts of healthy normotensive individuals not taking chronic medication. The obtained results (Tables 1 and 2) show visible night-to-day ratio variability in both groups, where the mean 7-day night-to-day ratio shows values different from those obtained for the individual days across the week period. Taking just single-day values (one 24-hour cycle) into account, the examined individuals could often be diagnosed incorrectly, showing false positive as well as false negative results of blood pressure testing with the risk level ranging from 0 to 100% (with the mean at 40% in men and 50% in women). This difference between healthy men and healthy women is statistically insignificant (0.490). However, the previous suggests that the mean value for 7 days (7-day ABPM) represents a more accurate method of specifying valid blood pressure and night-to-day ratio values as a prediction factor of the level of cardiovascular risk.

Furthermore, the next part of the study evaluating healthy subjects with controlled physical activity (Table 2) found that one of the tested individuals could be classified with the same category on the basis of an individual 24-hour cycle when compared to the whole-week evaluation. Four individuals showed 24-hour values closest to the 7-day mean, and therefore their classification remained the same after the 7-day testing as when based on the individual 24-hour test, with just one of the seven days showing a different result (86% accordance). This classification was also identical to the classification based on the mode night-to-day ratio for the week. In one case, the accordance rate reached 0%. According to the night-to-day ratio during 7 days, this subject (no. 24) was classified as nondipper, while the same result appeared in none of the 7 individually monitored days. In cases such as this, the usability of the night-to-day ratio seems problematic. Even though in three of the days the subject's data corresponded to the dipper category, in the remaining 4 days (mode) the subject appeared as a reverse dipper. This is the category with the highest predicted risk [11, 12]. There are also several studies with the 24-h record, that make clear that reverse dipping and nondipping had the worst outcome [22, 23]. The cohort of 51 patients under cardiovascular rehabilitation (group 4) included 3 individuals classified in all 7 individual days according to their night-to-day ratio profiles with the same category as according to the whole-week result (100% accordance). Three individuals showed 24-hour values closest to the 7-day mean, and therefore their classification remained the same after the 7-day testing as when based on the individual 24-hour test, with just one of the seven days showing a different result (86% accordance). In spite of this, considerable variability was observed in the remaining 45 patients, which, in the case of mere 24-hour monitoring, could lead to the incorrect prediction of cardiovascular risk or disease progression. The variability of the night-to-day ratio permits a more accurate specification of the prediction and prevalence of certain diseases, for example, cardiovascular disease, or the risk of occurrence of fatal, as well as nonfatal cardiovascular incidents [22–24]. Certain disadvantages of 24-h ABPM in comparison to 7-day ABPM due to the novelty effect were also pointed out by other studies [14, 25]. In contrast, even 24-h ABPM is sufficient to detect a progressive increase in cardiovascular events occurred from dipper to nondipper and reverse dipper participants [23]. Hermida et al. presented a study with a 48-h record and concluded the superiority of the 48-h ABPM record over the 24-h ABPM record. Reduction of ABPM duration to just 24-h resulted in an error in the determination of the most significant prognostic markers of cardiovascular events [26]. However, studies in hypertensive patients demonstrate that as many as 35% of hypertensive patients initially classified as nondippers did not confirm their nocturnal profile at a second ABPM recording and that remains when analysed with a period time of at least 5 months [27]. As the variability of the night-to-day ratio is not predicted by easily available clinical data, such as sex and age, a reliable classification of patients according to circadian blood pressure patterns should be obtained by repeating ABPM [28].

Insufficient blood pressure decrease during night sleep (nondippers) is certainly a significant predictive factor for mortality, but other coincident risk factors (sleep quality and sodium intake) must also be taken into account [8, 23, 29, 30]. There are also other studies emphasizing the significance of nocturnal blood pressure decrease as a predictor of death and cardiovascular incident occurrence in hypertonic patients [31]. Literature references also mention the increased variability of day blood pressure values in patients with depression [32].

The same applies to risk groups, i.e., seniors, diabetic patients, obese individuals, or pregnant women [29, 33]. The relationship has been described between a reduced drop in nocturnal blood pressure and total cardiovascular risk in patients with essential arterial hypertension [34]. The question is whether the specification of the night-to-day ratio of each individual for each day of the 7-day ABPM monitoring would be the most convenient option or not. In many patients, diagnosing could thus be based on the most frequently occurring values (mode specification). However, even here differences can be found in comparison with the results based on the mean blood pressure values for 7 days, as additionally shown in Tables 1–4. As each patient changes several patterns night-to-day ratio during the 7 days is difficult to define. It is questionable whether night-to-day ratio is not burdened by the inaccuracy of discontinuous distribution. There are other continuous methods. Pulse wave velocity (PWV) is used clinically as a measure of arterial stiffness and can be readily measured noninvasively in humans, with the measurement of carotid to femoral PWV (cfPWV) being the recommended method. cfPWV is highly reproducible and predicts future cardiovascular events and all-cause mortality independent of conventional cardiovascular risk factors. It has been recognized by the European Society of Hypertension as an indicator of target organ damage and a useful additional test in the investigation of hypertension [35]. Ambulatory arterial stiffness index (AASI) levels are associated with night-to-day blood pressure ratio. Lower levels of AASI are significantly associated with extreme dipper and dipper blood pressure nocturnal profile when compared to healthy controls. After correction for the major confounding factors, the association between AASI and the high-damaged class of hypertensive subjects with lower or no nocturnal fall of blood pressure is lost. AASI is unable to estimate arterial stiffness of older hypertensive subjects with a high burden of organ and vascular damage and several comorbidities, probably because the nocturnal reduction of blood pressure is the main determinant of AASI, being more powerful than arterial stiffness itself [36].

Night-to-day ratio specification for each day from 7-day ABPM also represents a doubtless advantage in that it allows for the recording of individual variability and frequency of occurrence of high-risk days. Discussions with the examined individuals can then find which particular activity of the subject caused a blood pressure response of the nondipper (ND), extreme dipper (ED), or reverse dipper (R) type.

Although the relationships between circadian blood pressure variability and physical activity are not yet quite clear, most studies dealing with this issue point to the positive effects of physical activity on the cardiovascular system and blood pressure values [37, 38].

According to the results of our study combined training does not change the variability of blood pressure (the night-to-day ratio was calculated from systolic blood pressure).

Due to lifestyle in the so-called “civilized” world, the percentage of the urban population with blood pressure higher than 140/90 mmHg or who uses antihypertensive drugs increases with age. The high prevalence of cardiovascular risk factors in populations is significantly associated with life conditions. Abnormal dipping patterns is important prognostic markers for future cardiovascular morbidity in resistant hypertension patients. Extreme dipping is a protective factor in younger individuals but a hazardous factor in the elderly and in individuals with previous cardiovascular diseases [39]. High cardiovascular risk is also associated with reverse dippers [23]. In patients with uncomplicated hypertension, the reproducibility of dipping is relatively low. Antihypertensive drug treatment can normalize the nondipping pattern, but the therapeutic consequences of this are unknown [8]. To improve cardiovascular prevention, an appropriate antihypertensive chronotherapeutic approach should be implemented for these different conditions [23].

In contrast, the findings in the isolated populations (Yanomami Indians from the Amazon rainforest and rural populations) showed that neither blood pressure elevation with age is inevitable, nor is the high prevalence of hypertension [40].

## 5. Conclusion

On the basis of the obtained results, the following procedure can be recommended for clinical practice. If the standard 24-hour monitoring is used, then obtain at least 3 mutually independent 24-hour cycle data for every examined individual. If at least two of them result in the same night-to-day ratio and the third night-to-day ratio is only slightly different, the thus obtained index can be considered trustworthy. Otherwise, the correct night-to-day ratio can only be obtained by 7-day ABPM and, in addition, the resulting night-to-day ratio calculated from the mean values obtained in the course of the 7 days must be compared to the result modes obtained for the individual monitoring days. Our own clinical experience teaches us that there are subjects whose accurate night-to-day ratio-based classification is difficult due to the high-value variability between days. Therefore, the conclusion can be drawn that the mean value of the night-to-day ratio found by ambulatory BP monitoring across 7 days is a much more accurate method of valid value specification when compared to 24-h ABPM. In the case of the individuals included in the monitored groups, the mean night-to-day ratio-based (mode for the 7 days versus the individual days of 24-hour monitoring) classification accordance ranged between 59% and 62%. Only in singular cases did the mean accordance reach 100%. The accordance size was not dependent on the gender, age, health, or cardiovascular disease, or physical activity of the monitored individuals.

## Figures and Tables

**Table 1:** Classification accordance (%) of night decrease of blood pressure based on the definition night-to-day ratio (D, ND, ED, and R) specified from the quotient of values for 7 days and values for individual days and mode of classification in the individual days in a cohort of 40 healthy subjects without exercise.

No	Mean	Night-to-day ratio (%)				Accordance	Mode	Accordance
	7 days	R	ND	D	ED	7 : 1 day	7 days	7 : 1 day
1	ND	14	57	29		57	ND	57
2	ND	17	33	50		33	D	50
3	ND	14	29	57		29	D	57
4	D	14	29	57		57	D	57
5	D		43	57		57	D	57
6	D		29	71		71	D	71
7	D	14	14	71		71	D	71
8	D		14	86		86	D	86
9	D		17	83		83	D	83
10	D		14	86		86	D	86
11	D		14	86		86	D	86
12	D		14	57	29	57	D	57
13	D	14		43	43	43	ED	43
14	D		14	43	43	43	D/ED	43
15	D		17	33	50	33	ED	50
16	D			71	29	71	D	71
17	D			71	29	71	D	71
18	D		14	29	57	29	ED	57
19	D		17	33	50	33	ED	50
20	ED		50	50		50	D/ED	50
21	ND	14	43	43		43	D/ND	43
22	ND	14	29	57		29	D	57
23	ND	14	29	57		29	D	57
24	ND	14	14	57	15	14	D	57
25	ND		43	43	14	43	D/ND	43
26	D		57	29	14	29	ND	57
27	D		29	71		71	D	71
28	D		29	71		71	D	71
29	D		15	71	14	71	D	71
30	D		29	57	14	57	D	57
31	D		43	29	28	29	ND	43
32	D			86	14	86	D	86
33	D		15	71	14	71	D	71
34	D		14	57	29	57	D	57
35	D	14	14	43	29	43	D	43
36	D			71	29	71	D	71
37	D			57	43	57	D	57
38	D			71	29	71	D	71
39	ED			43	57	57	ED	57
40	ED			29	71	71	ED	71
Mean						55		62
Min-max						29–86		43–86

D: dipper, ND: nondipper, ED: extreme dipper, and R: risers (reverse dipper). Mode: classification based on the highest frequency of category occurrence across individual days. /: mode cannot be clearly specified.

**Table 2:** Classification accordance (%) of night decrease of blood pressure based on the definition night-to-day ratio (D, ND, ED, R) specified from the quotient of values for 7 days and values for individual days and mode of classification in the individual days in a cohort of 40 healthy subjects with exercise.

No	Mean	Night-to-day ratio (%)				Accordance	Mode	Accordance
	7 days	R	ND	D	ED	7 : 1 day	7 days	7 : 1 day
1	ND		57	43		57	ND	57
2	D		50	50		50	ND/D	50
3	D		29	71		71	D	71
4	D	14	15	57	14	57	D	57
5	D	14	15	57	14	57	D	57
6	D		29	71		71	D	71
7	D	14		71	15	71	D	71
8	D	14	15	71		71	D	71
9	D		29	71		71	D	71
10	D		29	71		71	D	71
11	D		29	57	14	57	D	57
12	D		14	57	29	57	D	57
13	D		14	71	14	71	D	71
14	D			100		100	D	100
15	D		14	71	14	71	D	71
16	D		14	86		86	D	86
17	D		29	43	28	43	D	43
18	D		14	57	29	57	D	57
19	D		14	43	43	43	D/ED	43
20	D			29	71	29	ED	71
21	ND	43	43	14		43	ND/R	43
22	ND	29	57	14		57	ND	57
23	ND	33	50	17		50	ND	50
24	ND	57		43		0	R	57
25	ND	29	43	28		43	ND	43
26	ND	29	43	14	14	43	ND	43
27	ND	29	29	29	13	29	D/ND/R	29
28	ND		86	14		86	ND	86
29	ND		57	29	14	57	ND	57
30	ND		57	29	14	57	ND	57
31	D	29	13	29	29	29	D/ED/R	29
32	D		57	14	29	14	ND	57
33	D		43	29	28	29	ND	43
34	D		14	86		86	D	86
35	D		14	71	15	71	D	71
36	D		43	43	14	43	D/ND	43
37	D		14	86		86	D	86
38	D		29	43	28	43	D	43
39	D		28	43	29	43	D	43
40	ED			43	57	57	ED	57
Mean						56		60
Min-max						0–100		43–100

D: dipper, ND: nondipper, ED: extreme dipper, and R: risers (reverse dipper). Mode: classification based on the highest frequency of category occurrence across individual days. /: mode cannot be clearly specified.

**Table 3:** Classification accordance (%) of night decrease of blood pressure based on the definition night-to-day ratio (D, ND, ED, and R) specified from the quotient of values for 7 days and values for individual days and mode of classification in the individual days in a cohort of 40 patients without exercise.

No	Mean	Night-to-day ratio (%)				Accordance	Mode	Accordance
	7 days	R	ND	D	ED	7 : 1 day	7 days	7 : 1 day
1	ND	14	86			86	ND	86
2	ND	14	57	29		57	ND	57
3	ND	14	29	57		29	D	57
4	ND		86		14	86	ND	86
5	ND		57	43		57	ND	57
6	D		43	57		57	D	57
7	D	14	14	72		72	D	72
8	D		14	57	29	57	D	57
9	D		29	29	42	29	ED	42
10	D		14	57	29	57	D	57
11	D		14	43	43	43	D/ED	43
12	D		29	28	43	29	ED	43
13	D		14	29	57	29	ED	57
14	ED		14		86	86	ED	86
15	ED			57	43	43	D	57
16	ED		14	14	72	71	ED	71
17	ED			50	50	50	ND/D	50
18	ED			43	57	57	ED	57
19	ED		17	33	50	50	ED	50
20	ED			14	86	86	ED	86
21	ND	57	29	14		29	RD	57
22	ND	29	71			71	ND	71
23	ND	14	57	29		57	ND	57
24	ND	14	57	29		57	ND	57
25	ND		57	43		57	ND	57
26	ND	14	43	43		43	ND/D	43
27	D		72	14	14	14	ND	72
28	D		57	29		14	ND	57
29	D	14	14	72		72	D	72
30	D		43	43	14	43	ND/D	43
31	D		29	71		71	D	71
32	D		43	43	14	43	ND/D	43
33	D		29	43	28	43	D	43
34	D		14	57	29	57	D	57
35	D		14	57	29	57	D	57
36	D		29	43	28	43	D	43
37	D			57	43	57	D	57
38	D			57	43	57	D	57
39	D			43	57	43	ED	57
40	ED			29	71	71	ED	71
Mean						53		59
Min-max						29–86		43–86

D: dipper, ND: nondipper, ED: extreme dipper, and R: risers (reverse dipper). Mode: classification based on the highest frequency of category occurrence across individual days. /: mode cannot be clearly specified.

**Table 4:** Classification accordance (%) of night decrease of blood pressure based on the definition night-to-day ratio (D, ND, ED, and R) specified from the quotient of values for 7 days and values for individual days and mode of classification in the individual days in a cohort of 51 patients with exercise.

No	Mean	Night-to-day ratio (%)				Accordance	Mode	Accordance
	7 days	R	ND	D	ED	7 : 1 day	7 days	7 : 1 day
1	R	71	29			71	R	71
2	ND	43	57			57	ND	57
3	ND	67	33			33	R	67
4	ND	29	71			71	ND	71
5	ND	43	57			57	ND	57
6	ND	43	29	28		29	R	43
7	ND	28	43	29		43	ND	43
8	ND	57	14	29		14	R	57
9	ND	29	43	28		43	ND	43
10	ND	29	57	14		57	ND	57
11	ND	28	29	43		29	D	43
12	ND		86	14		86	ND	86
13	ND	29	43	28		43	ND	43
14	ND	14	43	43		43	D/ND	43
15	ND	14	43	43		43	D/ND	43
16	ND	14	57	15	14	57	ND	57
17	ND	14	43	43		43	D/ND	43
18	ND	15	57	14	14	57	ND	57
19	ND	14	43	43		43	D/ND	43
20	ND		57	29	14	57	ND	57
21	ND		71	15	14	71	ND	71
22	D		57	43		43	D	57
23	D		57	43		43	D	57
24	D		43	43		43	D/ND	43
25	D		57	29	14	29	ND	57
26	D		43	43	14	43	D/ND	43
27	D	14	14	71		71	D	71
28	D		57	43		43	ND	57
29	D	13	29	29	29	29	D/ND/ED	29
30	D	15		71	14	71	D	71
31	D	29	13	29	29	29	D/ED/R	29
32	D			100		100	D	100
33	D		43	43	14	43	D/ND	43
34	D	13	29	29	29	29	D/ND/ED	29
35	D		14	71	14	71	D	71
36	D		29	57	14	57	D	57
37	D		14	86		86	D	86
38	D		33	50	17	50	D	50
39	D			100		100	D	100
40	D	14	15	14	57	14	ED	57
41	D		15	71	14	71	D	71
42	D		29	43	28	43	D	43
43	D		14	71	15	71	D	71
44	D			71	29	71	D	71
45	D			71	29	71	D	71
46	D			43	57	43	ED	57
47	D			50	50	50	D/ED	50
48	ED		14	29	57	57	ED	57
49	ED			29	71	71	ED	71
50	ED			14	86	86	ED	86
51	ED				100	100	ED	100
Mean						54		59
Min-max						14–100		29–100

D: dipper, ND: nondipper, ED: extreme dipper, and R: risers (reverse dipper). Mode: classification based on the highest frequency of category occurrence across individual days. /: mode cannot be clearly specified.

## Data Availability

Data are available upon request.
